# Free‐Breathing Functional Pulmonary Proton MRI: A Novel Approach Using Voxel‐Wise Lung Ventilation (VOLVE) Assessment in Healthy Volunteers and Patients With Chronic Obstructive Pulmonary Disease

**DOI:** 10.1002/jmri.29444

**Published:** 2024-05-31

**Authors:** Zachary J.T. Peggs, Jonathan P. Brooke, Charlotte E. Bolton, Ian P. Hall, Susan T. Francis, Penny A. Gowland

**Affiliations:** ^1^ Sir Peter Mansfield Imaging Centre, School of Physics and Astronomy University of Nottingham Nottingham UK; ^2^ Centre for Respiratory Research NIHR Nottingham Biomedical Research Centre Nottingham UK; ^3^ Centre for Respiratory Research, Translational Medical Sciences, School of Medicine University of Nottingham Nottingham UK; ^4^ Department of Respiratory Medicine Nottingham University Hospitals NHS Trust Nottingham UK

**Keywords:** lung, pulmonary, ventilation, perfusion, COPD

## Abstract

**Background:**

In respiratory medicine, there is a need for sensitive measures of regional lung function that can be performed using standard imaging technology, without the need for inhaled or intravenous contrast agents.

**Purpose:**

To describe VOxel‐wise Lung VEntilation (VOLVE), a new method for quantifying regional lung ventilation (V) and perfusion (Q) using free‐breathing proton MRI, and to evaluate VOLVE in healthy never‐smokers, healthy people with smoking history, and people with chronic obstructive pulmonary disease (COPD).

**Study Type:**

Prospective pilot.

**Population:**

Twelve healthy never‐smoker participants (age 30.3 ± 12.5 years, five male), four healthy participants with smoking history (>10 pack‐years) (age 42.5 ± 18.3 years, one male), and 12 participants with COPD (age 62.8 ± 11.1 years, seven male).

**Field Strength/Sequence:**

Single‐slice free‐breathing two‐dimensional fast field echo sequence at 3 T.

**Assessment:**

A novel postprocessing was developed to evaluate the MR signal changes in the lung parenchyma using a linear regression‐based approach, which makes use of all the data in the time series for maximum sensitivity. V/Q‐weighted maps were produced by computing the cross‐correlation, lag and gradient between the respiratory/cardiac phase time course and lung parenchyma signal time courses. A comparison of histogram median and skewness values and spirometry was performed.

**Statistical Tests:**

Kruskal–Wallis tests with Dunn's multiple comparison tests to compare VOLVE metrics between groups; Spearman correlation to assess the correlation between MRI and spirometry‐derived parameters; and Bland–Altman analysis and coefficient of variation to evaluate repeatability were used. A *P*‐value <0.05 was considered significant.

**Results:**

Significant differences between the groups were found for ventilation between healthy never‐smoker and COPD groups (median XCC_V_, Lag_V_, and Grad_V_) and perfusion (median XCC_Q_, Lag_Q_, and Grad_Q_). Minimal bias and no significant differences between intravisit scans were found (*P* range = 0.12–0.97).

**Data Conclusion:**

This preliminary study showed that VOLVE has potential to provide metrics of function quantification.

**Level of Evidence:**

2

**Technical Efficacy:**

Stage 1

The proliferation of personalized therapies in respiratory medicine has created a clinical need for new pulmonary diagnostics that provide highly sensitive measures of regional lung function.^1^

Since the development of free‐breathing dynamic proton MRI using Fourier decomposition (FD) by Bauman et al in 2009,^2^ there has been substantial progress in FD‐MRI and related methods.^3^, ^4^, ^5^, ^6^, ^7^ The FD‐MRI method exploits the fact that both the respiratory and cardiac cycles give rise to periodic signal changes in the lung that can be detected on rapid dynamic sequences of MRI scans. This enables spectral separation of the signals via Fourier analysis, yielding information related to both ventilation and perfusion. Successive modifications to the FD technique,^3^, ^4^, ^5^ related approaches using self‐gating,^6^ and more recently, full respiratory and cardiac cycle reconstruction via retrospective image sorting^7^ have all shown success in producing maps of pulmonary function with standard MRI technology.

A key metric of ventilation‐weighted information from these techniques is fractional ventilation (FV). This was first introduced by Zapke et al and is a measure of the fractional change in lung tissue signal with respiration.^8^ In recent studies,^3^, ^7^ FV has been defined as:

(1) $$\mathrm{FV}=\frac{{\bar{\mathrm{SI}}}_{\exp}-{\bar{\mathrm{SI}}}_{\text{insp}}}{{\bar{\mathrm{SI}}}_{\exp}}$$

where ${\bar{\mathrm{SI}}}_{\exp}$ and ${\bar{\mathrm{SI}}}_{\text{insp}}$ are the image signal intensities (SI) at expiration and inspiration, respectively, averaged over respiratory cycles. Averaging minimizes the effect of the variation in blood volume between cycles, limits the effects of variations across the respiratory cycle (including variations in respiration amplitude), and compensates for temporal undersampling of the respiratory cycle.^3^ To calculate FV, images from expiration and inspiration must be identified by sorting data according to respiratory phase; several approaches have been proposed for this retrospective sorting.^6^, ^7^, ^9^ Other ventilation metrics also exist, including specific ventilation, SV = (*S*_exp_ − *S*_insp_)/*S*_insp_,^10^, ^11^ and regional ventilation, RVent = (*S*_mid_/*S*_insp_) − (*S*_mid_/*S*_exp_),^12^ which each use different respiratory cycle phases for ventilation quantification. Related analyses have been performed using the Jacobian determinant from image registration as a measure of local lung expansion.^3^

The principal drawback of static FV‐related approaches, in which calculation of the ventilation parameters involves only two or three respiratory states, is their dependence on the exact breathing behavior exhibited when the data is acquired. Since FV depends on the signal at end‐expiration and end‐inspiration (Eq. 1), any variation in tidal volume inevitably leads to variation in the measured FV.^13^ Thus variability in the amplitude of breathing, especially across different patient groups, makes characterization of ventilation via FV challenging. Voskrebenzev et al introduced a correction for this effect, utilizing the lung area time series derived from the inverse deformation fields from image registration to determine a tidal volume adjustment, which was found to improve FV repeatability and reproducibility.^14^ However, the production of static ventilation maps still uses the data inefficiently and introduces bias based on the amplitudes of the respiratory cycle phases used in the analysis. In addition, quantification of perfusion‐related measures is confounded by the need for a reference signal from a completely blood‐filled voxel, such as from the aorta.^15^ As noted by Glandorf et al, this is problematic due to the large variation in the signal within the lumen of the aorta, making alternative quantification methods desirable.^16^

The Phase‐REsolved FUnctional Lung (PREFUL) technique has been used to determine dynamic regional flow‐volume parameters (imaging‐based spirometry), rather than the static ventilation measures derived from previous analyses.^17^ These dynamic assessments are achieved by voxel‐wise determination of the slope of RVent(*t*) = (*S*_exp_‐*S*(*t*))/*S*_exp_, which gives a surrogate for airflow. These regional flow‐volume loops can be transformed into functional maps by comparing each loop to a healthy reference loop via cross‐correlation. The assessment of ventilation dynamics via regional flow‐volume loops has been shown to correlate with FEV_1_ in chronic obstructive pulmonary disease (COPD) and be more sensitive than static ventilation measures in early chronic lung allograft dysfunction.^18^ However, this technique still assumes a cosine model for sorting the images into a canonical respiratory cycle, and quantification requires the determination of a healthy reference lung region‐of‐interest (ROI) for cross‐correlation comparison.

Thus the aim of this study was to develop a linear regression‐based analysis pipeline of a standard fast field echo (FFE) dynamic MRI time series from which information relating to ventilation could be determined that is independent of breathing behavior; this is termed VOxel‐wise Lung VEntilation (VOLVE). A secondary aim was to extend the method to assess perfusion‐weighted signal changes in the lungs.

## Methods

### Theory

**Figure 1:**
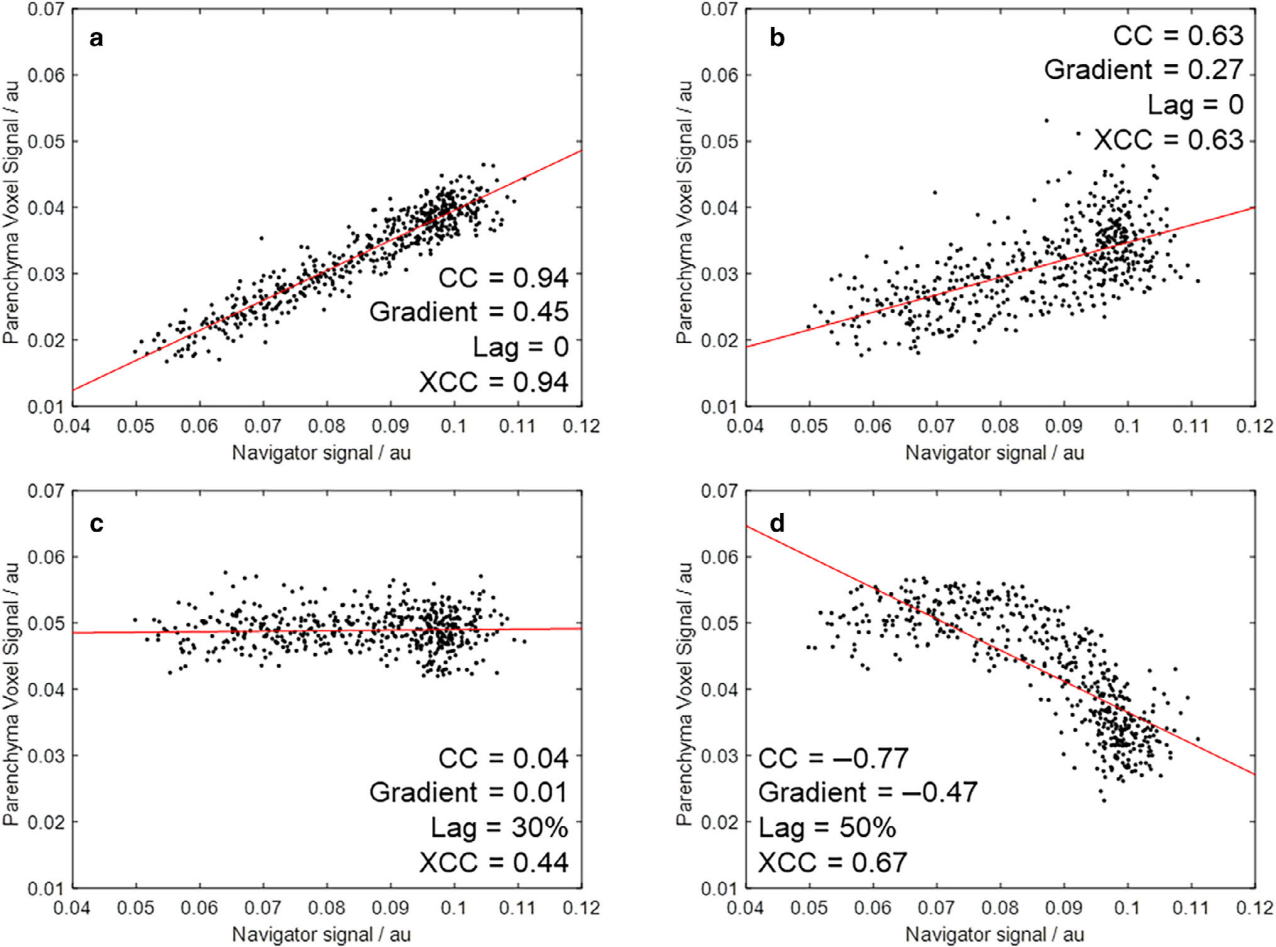
Example scatter plots of parenchyma versus navigator signals across the image time series for a healthy participant. Four voxels with varying correlation coefficients (CC) are shown to illustrate (**a**) strong correlation, (**b**) moderate correlation, (**c**) poor correlation, and (**d**) negative correlation. Note that some lung parenchyma voxels display nonlinear relationships, as shown in (**d)**. Voxels (a) and (b) have zero lag (and hence the cross‐correlation (XCC) is equal to the CC), whereas voxels (c) and (d) are lagged by 30% (110° phase shift) and 50% (180° phase shift) of the respiratory cycle, respectively.

In lung imaging approaches that require image‐resorting such as PREFUL,^7^ functional parameters are calculated by collapsing the image series into a single respiratory cycle from which the fractional signal change between respiratory phases is estimated. VOLVE uses an alternative approach, regressing the MR signal of the entire image series against respiratory phase. Two time series signals are extracted directly from the dynamic image series for analysis: 1) the lung parenchyma signal per voxel, and 2) a global navigator signal based on the position of the diaphragm that indicates the relative phase of the respiratory cycle, with the correlation between these investigated. To account for the fact that some voxels exhibit a delayed (lagged) signal variation, the cross‐correlation (XCC) is used, as illustrated in Fig. 1.

Voxel‐wise lung parenchyma signal time courses are extracted following nonrigid registration of the image series and segmentation of the lung parenchyma. The navigator signal, which is the same for all voxels, is determined by calculating the mean signal inside an ROI across the lung–diaphragm boundary, following previous FD‐based methods of analysis.^19^ The amplitude of the navigator signal has local maxima at end‐expiration and local minima at end‐inspiration.

Assuming a linear relationship, a voxel‐wise linear regression is performed between the parenchyma and navigator signals, and the resulting gradient used to estimate the effect of phase of respiration on lung tissue density. The gradient provides information that is analogous to FV, but importantly utilizes images from across the entire respiratory cycle, rather than being driven by those at only end‐expiration/inspiration. The proposed methodology avoids the need to choose predefined points in the respiratory cycle for ventilation quantification, mitigating against the confounding effects of variations in levels of ventilation between scans. Instead, in VOLVE all respiratory states contribute to the assessment of the periodic changes in the MR signal in the lung parenchyma and provide a metric of functional assessment. Additional quantities such as the cross‐correlation (XCC) and associated lag can be determined by iteratively shifting the time courses with respect to one another, and importantly, nonlinearities explored (as shown in Fig. 1d).

In VOLVE analysis, as there are errors in both the lung parenchyma and navigator signals, standard least squares fitting approaches are not appropriate in fitting the gradient. Instead the linear relationship is characterized using York regression, where the least‐square estimation expressions are evaluated at the least‐square‐adjusted points rather than at the observed points.^20^ Full details are provided in York et al.^21^ This allows the gradient to be determined more accurately, even in the presence of a phase shift between the navigator and signal time series (see Fig. S1 in Data S1).

### Study Participants and MRI Protocol

This study was approved by the relevant ethics committees and all subjects provided written informed consent.

To investigate the use of VOLVE analysis, 12 healthy participants with no tobacco smoking history or lung disease/symptoms, four healthy participants with >10 pack‐year smoking history but no respiratory symptoms nor evidence of airflow limitation or abnormality on spirometry (referred to henceforth as healthy smokers), and 12 participants with COPD (symptoms consistent with COPD, FEV_1_/FVC ratio <0.7 and >10 pack‐years of tobacco smoking) were recruited. Healthy never‐smokers were recruited under a University of Nottingham Faculty of Medicine and Health Sciences local research ethical approval. Participants with COPD and healthy smokers were recruited from either a complex COPD NHS outpatient clinic or from a COPD register, held within the NIHR Nottingham Biomedical Research Centre following research ethics approval (Wales REC 4; ethics reference 21/WA/0266). Retrospective spirometry data (FEV_1_ [% predicted], FVC [% predicted], and FEV_1_/FVC) were available for the healthy smokers and COPD participants, with measures taken within 24 months of study visits (mean ± standard deviation time between spirometry and MRI examination 10 ± 14 months).

MR images were acquired in a wide‐bore 3 T Ingenia MR scanner (Philips Medical Systems, Best, The Netherlands) using the FlexCoverage anterior coil with 32 channels and inbuilt posterior receive coils. The acquisition was carried out during free‐breathing using a two‐dimensional FFE sequence with parameters of TE/TR = 0.57/1.9 msec, flip angle = 18°, slice thickness 15 mm, field of view = 475 × 475 mm, matrix = 240 × 240, phase partial Fourier = 0.75, and bandwidth 1532 Hz, with no parallel acceleration. While the flip angle used was greater than the Ernst angle of ~3.5°, this was chosen to maintain a strong ventilation signal without suppressing the perfusion signal.^22^ A single coronal slice placed approximately 1 cm posterior to the heart, at the level of the descending aorta, was imaged repeatedly with a temporal resolution of 229 msec. This slice position was chosen to ensure minimal interference from cardiac motion and major pulmonary vessels, while capturing a large lung cross‐section. The sampling rate was required to avoid aliasing of the cardiac signals. A total of 512 dynamic images were acquired in 117 seconds, and collection of the dataset was repeated approximately 15 minutes later with the participant in approximately the same position (no repositioning or removal from scanner).

### VOLVE Analysis

VOLVE analysis was performed on the dynamic, single‐slice, FFE image time series, using software written in‐house in MATLAB R2022b (MathWorks, Natick, MA, USA). The global navigator signal time course and the voxel‐wise lung parenchyma time courses were determined having first removed the initial 15 images in the dataset to ensure the signal had achieved steady state.

The navigator signal was generated by calculating for each image in the time series the mean signal inside a rectangular ROI spanning the right hemidiaphragm to capture the apical‐basal diaphragmatic motion; the right lung was used to avoid contamination from cardiac signals. This ROI (30 voxels in foot‐head direction by five voxels in right–left direction) was defined automatically; the right lung was segmented via seeded region growing,^23^ and the ROI was centered on the right hemidiaphragm at one third of the distance between the costophrenic and costocardiac angles. The relative navigator signal (percentage of the median navigator signal) was computed as a proxy for relative lung volume change. Any extreme outliers in the navigator signal time series (more than three scaled median absolute deviations = median(|*X*_*i*_ – median(*X*)|), from its median) were removed, and a standard low‐pass filter (0.6 Hz) was applied to remove cardiac signal contributions.^4^

To obtain voxel‐wise lung parenchyma signal time courses, the single coronal slice FFE image time series were registered using a nonrigid demons‐based registration algorithm^24^ to a target image acquired close to the mean respiratory phase, i.e., at an intermediate level of lung inflation to minimize the need for large deformation fields.

Next, a lung mask was created for the registration target/fixed image using automatic segmentation of the thoracic cavity via seeded region growing, excluding large pulmonary vessels, and with manual correction if required (by Z.J.T.P., scientist with 3 years' experience in lung MR imaging). The lung parenchyma segmentations were then checked by a respiratory clinician (J.P.B., 4 years' experience in lung MR imaging). The registered parenchyma time series for voxels inside the lung mask were low‐pass filtered at 0.6 Hz to remove cardiac signal contributions (using the same filter as applied to the navigator signal).

**Figure 2:**
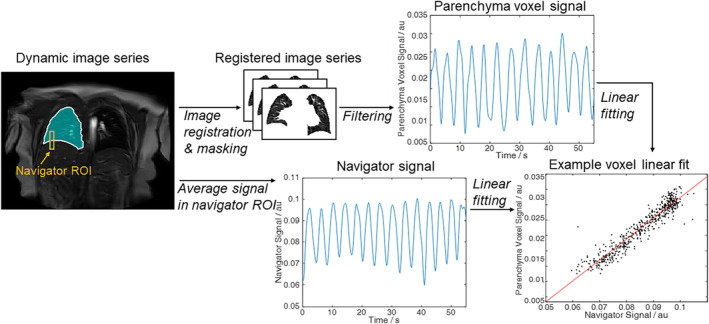
Overview of the image processing pipeline for VOLVE analysis. The signal in the lung–diaphragm ROI is averaged to generate the navigator signal over the image time series. Linear York regression is carried out voxel‐wise between this navigator signal and the registered lung parenchyma signal.

Finally, the lung parenchyma signals were compared to the navigator (respiratory phase) signals across the entire time course for each voxel in turn, computing ventilation (V) measures of 1) XCC_V_ and Lag_V_ maps—showing the maximum cross‐correlation and lag at which the maximum cross‐correlation occurred, and 2) Grad_V_ maps—showing the gradient of linear regression calculated via York regression. Lag_V_ was expressed as a percentage of the respiratory cycle having multiplied the lag in time by the average respiratory frequency determined by Fourier analysis of the navigator signal. An outline of the image processing pipeline is shown in Fig. 2.

**Figure 3:**
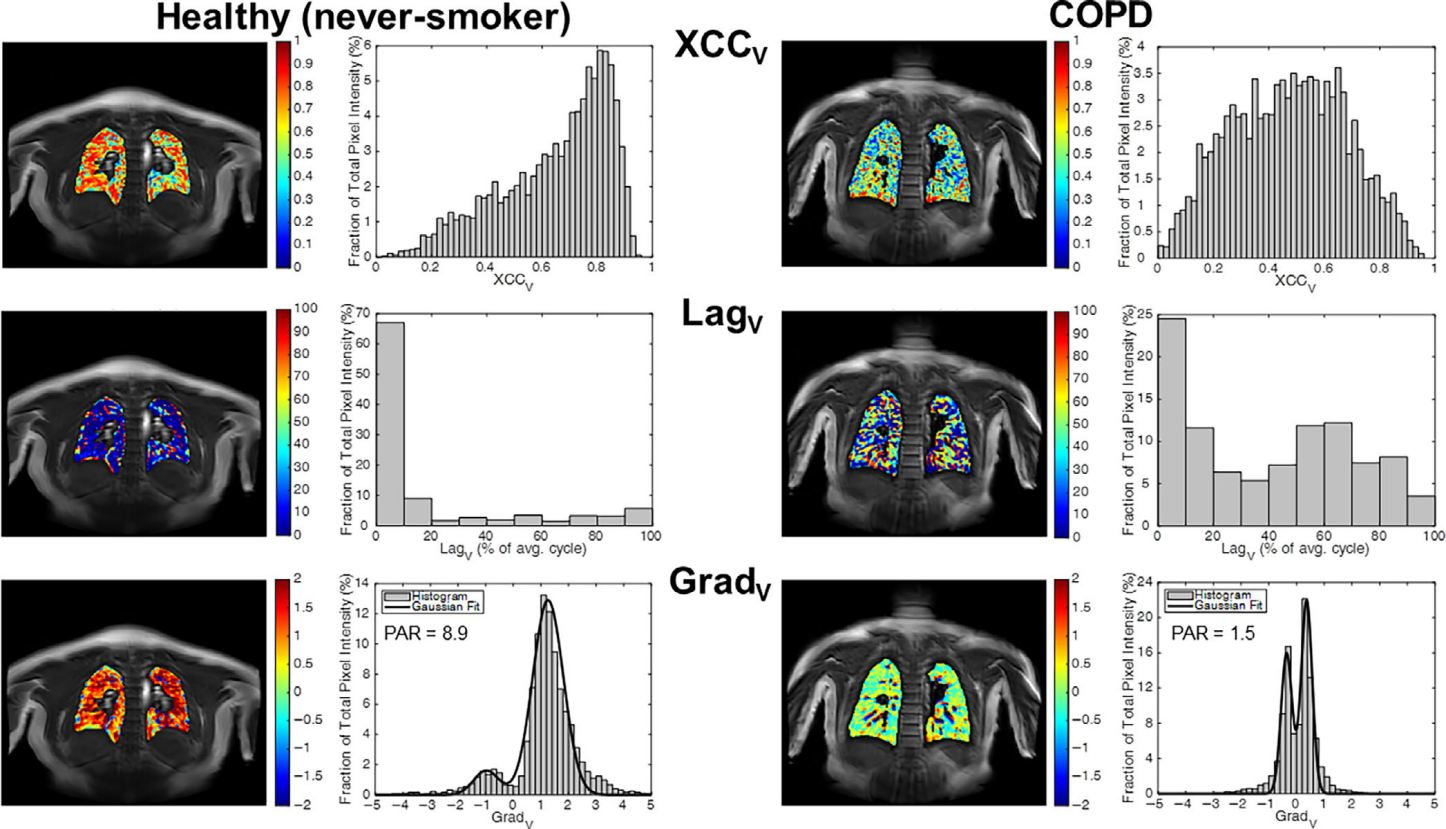
Example VOLVE parameter maps and corresponding histograms. Maximum cross‐correlation (XCC_V_) maps and histograms (top row); Lag_V_ maps and histograms (middle row); gradient (Grad_V_) maps and histograms fit with a Gaussian mixture model (bottom row); for a healthy never‐smoker (age 24, male) and COPD participant (age 66, female, FEV_1_ = 30%, GOLD III).

Histograms were created of the XCC_V_, Lag_V_, and Grad_V_ maps. The skewness of the XCC_V_ histogram was computed, and as the Grad_V_ histogram tended to be bimodal, this was fitted to a two‐component Gaussian mixture model and the peak area ratio (PAR) of the two fitted Gaussians was computed (see Fig. 3). The lung mask was also split into quadrants to explore regional analysis of measures. This quadrant segmentation was performed automatically using a similar algorithm to that used to determine the lung–diaphragm ROI, but in this case finding the midpoint between the apex and base of the right lung.

Image analysis was completed automatically without operator input, apart from any required manual corrections to the lung mask segmentation. Processing time for a single dataset containing 497 images (240 × 240 pixels) took approximately 1 minute on inexpensive hardware (AMD Ryzen 5 5600X CPU 3.7 MHz, 16 GB RAM), with image registration (utilizing parallelization) accounting for ~90% of this time.

### Assessment of Lung Perfusion

While the primary focus of this work was ventilation assessment, the same linear regression‐based framework could be applied to assess perfusion (Q) with only minor modifications to the analysis pipeline. By replacing the lung–diaphragm respiratory navigator signal with an aorta or main pulmonary vessel‐based cardiac signal time course (characterizing cardiac phase) and applying a high‐pass filter (0.8 Hz), VOLVE analysis was performed to yield perfusion information. This resulted in XCC_Q_, Lag_Q_, and Grad_Q_ maps, where for this analysis, Lag_Q_ is a measure of blood arrival time and so is shown as absolute time (in msec) rather than % of cardiac cycle.

### Statistical Analysis

Statistical analysis was carried out using GraphPad Prism 9.2.0 (GraphPad Software, San Diego, CA, USA). The VOLVE parameter data and spirometry data were found to be non‐normally distributed using a Shapiro–Wilk test, and hence nonparametric tests (Kruskal–Wallis test and Mann–Whitney *U* test) were used. Results were considered statistically significant for *P* < 0.05.

Comparison of VOLVE metrics between the healthy never‐smoker, healthy smoker, and COPD groups was performed using a Kruskal–Wallis test followed by post hoc pairwise analysis with Dunn's correction for multiple comparisons. Comparison of spirometry measures between healthy smokers and COPD patients was performed using a Mann–Whitney *U* test. Linear correlation between spirometry‐derived and MRI‐derived parameters was performed using the Spearman correlation coefficient.

To assess intravisit, intraparticipant repeatability, the coefficient of variation (CoV) was computed for all VOLVE metrics. Bland–Altman analysis was performed and the mean difference and 95% limits of agreement (mean ± 1.96 × standard deviation) was calculated. Repeat values were tested for significance via a Wilcoxon‐signed rank test.

## Results

### VOLVE: Ventilation Assessment

The MRI protocol was completed by all study participants. A summary of participant demographics and spirometry parameters is provided in Table 1.

**Table 1:** Participant Demographics and Spirometry Data

Parameter	Healthy (Never Smoker)	Healthy (Smoking History)	COPD	*P*‐Value		
				Healthy Never‐Smoker vs. Healthy Smoker	Healthy Never‐Smoker vs. COPD	Healthy Smoker vs. COPD
*N*	12	4	12	—	—	—
Sex (M:F)	5:7	1:3	7:5	—	—	—
Age (years)	30.3 ± 12.5	42.5 ± 18.3	62.8 ± 11.1	0.80	0.0009*	0.19
GOLD class						
1	—	—	0	—	—	—
2	—	—	4	—	—	—
3	—	—	7	—	—	—
4	—	—	1	—	—	—
FEV_1_ (L/minute)	NA	4.2 ± 1.2	1.3 ± 0.5	—	—	0.004*
FEV_1_% predicted	NA	106 ± 9	45.8 ± 15	—	—	0.004*
FVC (L/minute)	NA	5.1 ± 1.5	3.0 ± 0.9	—	—	0.031*
FVC % predicted	NA	103 ± 8	86 ± 17	—	—	0.10
FEV_1_/FVC	NA	0.82 ± 0.01	0.42 ± 0.09	—	—	0.002*

Values quoted as mean ± standard deviation, significant differences (*P* < 0.05) are marked with an asterisk (*).

M/F = male/female; GOLD = Global Initiative for Chronic Obstructive Lung Disease; FEV_1_ = forced expiratory volume in 1 second as a percentage of the predicted value; FVC = forced vital capacity; NA = not available.

Example XCC_V_, Lag_V_, and Grad_V_ maps are shown for a healthy never‐smoker and a COPD participant in Fig. 3. The XCC_V_ map for the healthy never‐smoker participant is relatively homogenous with XCC values generally >0.4 (histogram negatively skewed; skewness = −0.78), except for some small regions in, or bordering, vessels. In contrast, the XCC_V_ map for the COPD participant is heterogeneous (histogram symmetrically distributed with a skewness = −0.02). VOLVE maps for all study participants are provided in Supplementary Information S2.

The Lag_V_ map for the healthy never‐smoker participant shows that lags <20% of the respiratory cycle are predominant, with 68% of lung parenchyma voxels having a lag <10%. In contrast the Lag_V_ map for the COPD participant is heterogeneous with many voxels exhibiting a lag between 40% and 70% of the respiratory cycle. These voxels are out of phase with the navigator signal and indicate an *increase* in MR parenchyma signal on inspiration.

The Grad_V_ map for the healthy never‐smoker displays values generally >0.5, with the histogram showing a primary peak at 1, and a small secondary peak at −1. In contrast, the Grad_V_ map for the COPD participant shows a compressed distribution, with a higher proportion of zero values and a heightened secondary/negative peak, leading to a reduced PAR.

**Figure 4:**
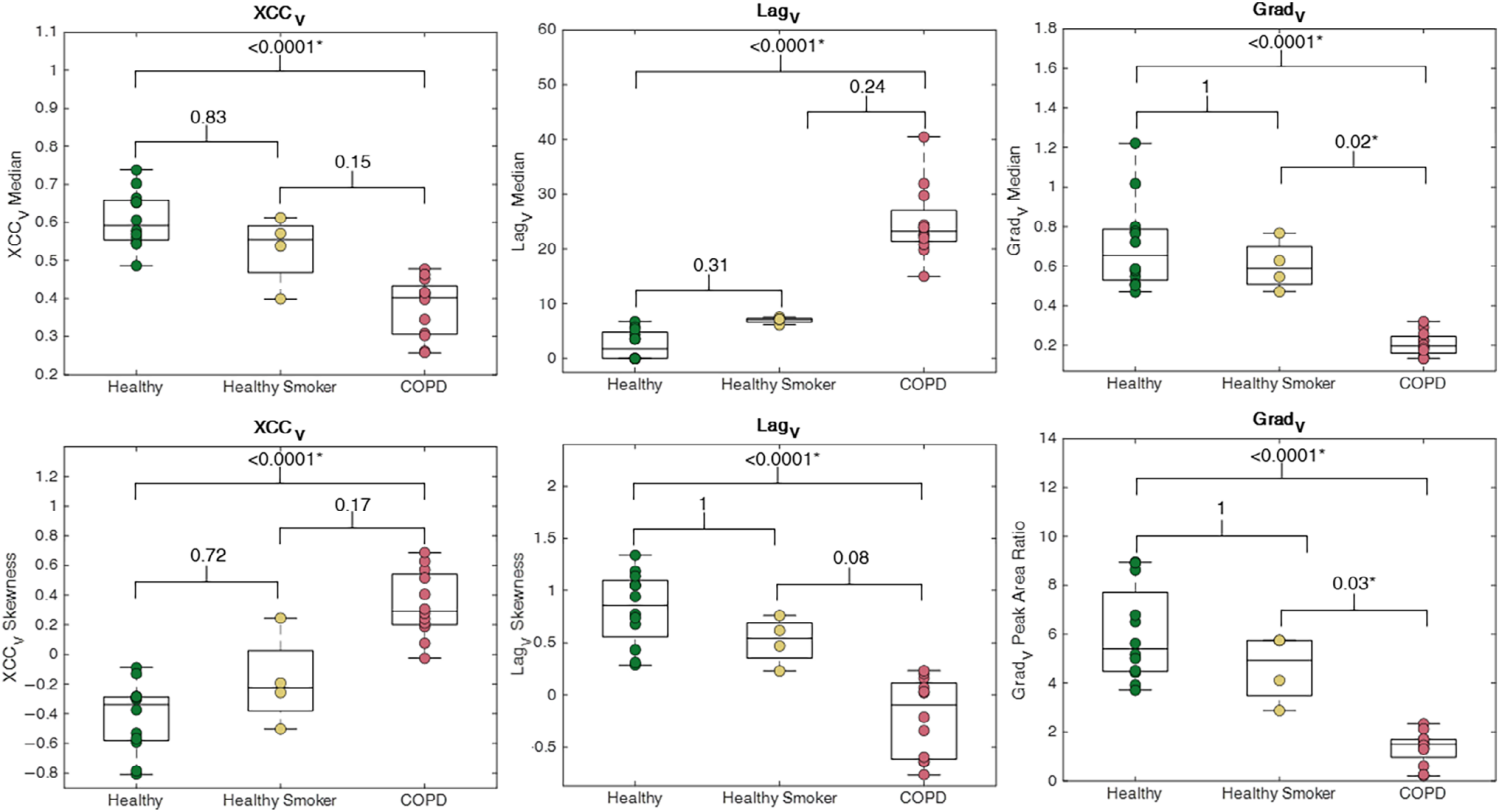
Boxplots showing the median and skewness for ventilation cross‐correlation (XCC_V_) and lag (Lag_V_), and median and fitted peak area ratio (PAR) for gradient (Grad_V_). Significant differences (*P* < 0.05*, Kruskal–Wallis test with Dunn's post hoc analysis) between COPD patients and both healthy participant groups (never‐smokers and those with smoking history) are observed for the Grad_V_ metrics, and between COPD and healthy never‐smokers for XCC_V_ and Lag_V_ metrics.

Figure 4 shows boxplots of the XCC_V_, Lag_V_, and Grad_V_ for the three groups. Grad_V_ metrics were significantly different in the COPD group compared to both the healthy never‐smoker group and the healthy smoker group. Both the XCC_V_ and Lag_V_ were significantly different between COPD and healthy never‐smokers.

**Figure 5:**
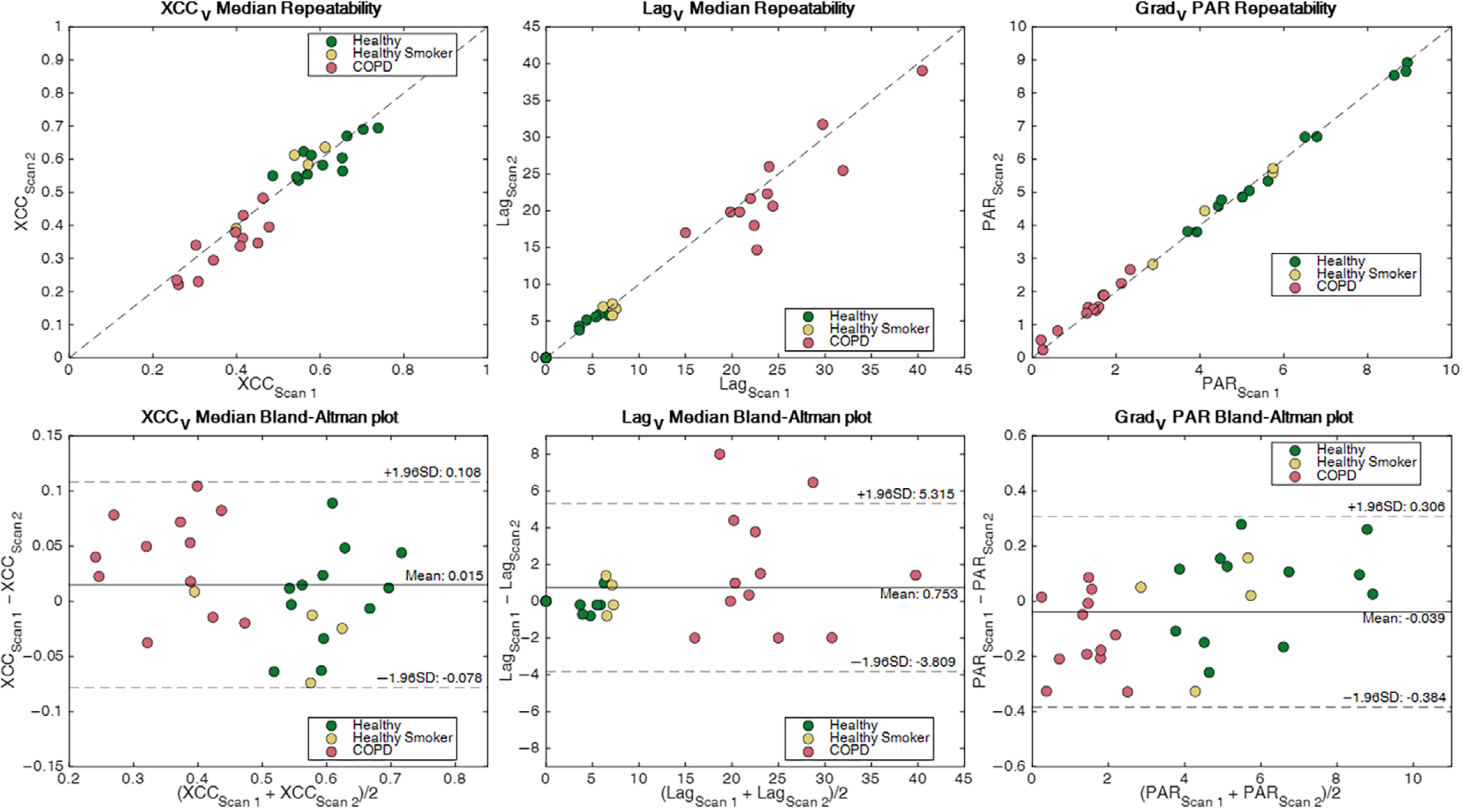
Repeatability assessment of VOLVE metrics (median XCC_V_, median Lag_V_, and Grad_V_ peak area ratio (PAR)). Plots of repeat values and Bland–Altman plots provide an assessment of within‐participant repeatability.

Figure 5 shows the within‐visit repeatability of the XCC_V_, Lag_V_, and Grad_V_ metrics and Bland–Altman plots. Bland–Altman analysis showed a mean difference of 0.015 (95% confidence interval (CI): ±0.093) for XCC_V_ median, 0.753 (95% CI: ±4.56) for Lag_V_ median, and −0.039 (95% CI: ±0.345) for Grad_V_ PAR. The median CoV across groups was 4.9% for XCC_V_ 7.1% for Lag_V_ and 2.3% for Grad_V_ PAR. No significant differences were found between intravisit scans (all *P* > 0.05, *P* range = 0.12–0.97; see Table S3 in Data S1).

Table 2 shows that the spirometric measures (FEV_1_ and FEV_1_/FVC) significantly correlated with VOLVE metrics (Lag_V_ and Grad_V_) across the healthy smoker and COPD groups. Moderate to weak correlations were observed for XCC_V_, but these were not significant (*P* range = 0.07–0.73) apart from skewness with FEV_1_/FVC.

**Table 2:** Spearman Correlations of VOLVE Metrics Against Standard Spirometry‐Derived Lung Function Metrics Given as *r* (*P*)

	FEV_1_	FVC	FEV_1_/FVC
XCC_V_ median	0.31 (0.20)	0.10 (0.73)	0.48 (0.07)
XCC_V_ skewness	−0.48 (0.07)	−0.26 (0.35)	−0.62 (0.02)*
Lag_V_ median	−0.49 (0.04)*	−0.22 (0.43)	−0.45 (0.10)
Grad_V_ median	0.64 (0.04)*	0.47 (0.08)	0.44 (0.01)*
Grad_V_ PAR	−0.79 (0.004)*	−0.52 (0.1)	−0.71 (0.01)*

Spirometry data (which was acquired only in healthy smoker and COPD participants) of FEV_1_ % predicted, FVC % predicted, and the ratio FEV_1_/FVC are shown, where significant correlations are marked with an asterisk (*).

FEV_1_ = forced expiratory volume in 1 second as a percentage of the predicted value; FVC = forced vital capacity; XCC = cross‐correlation; grad = gradient; PAR = peak area ratio.

**Figure 6:**
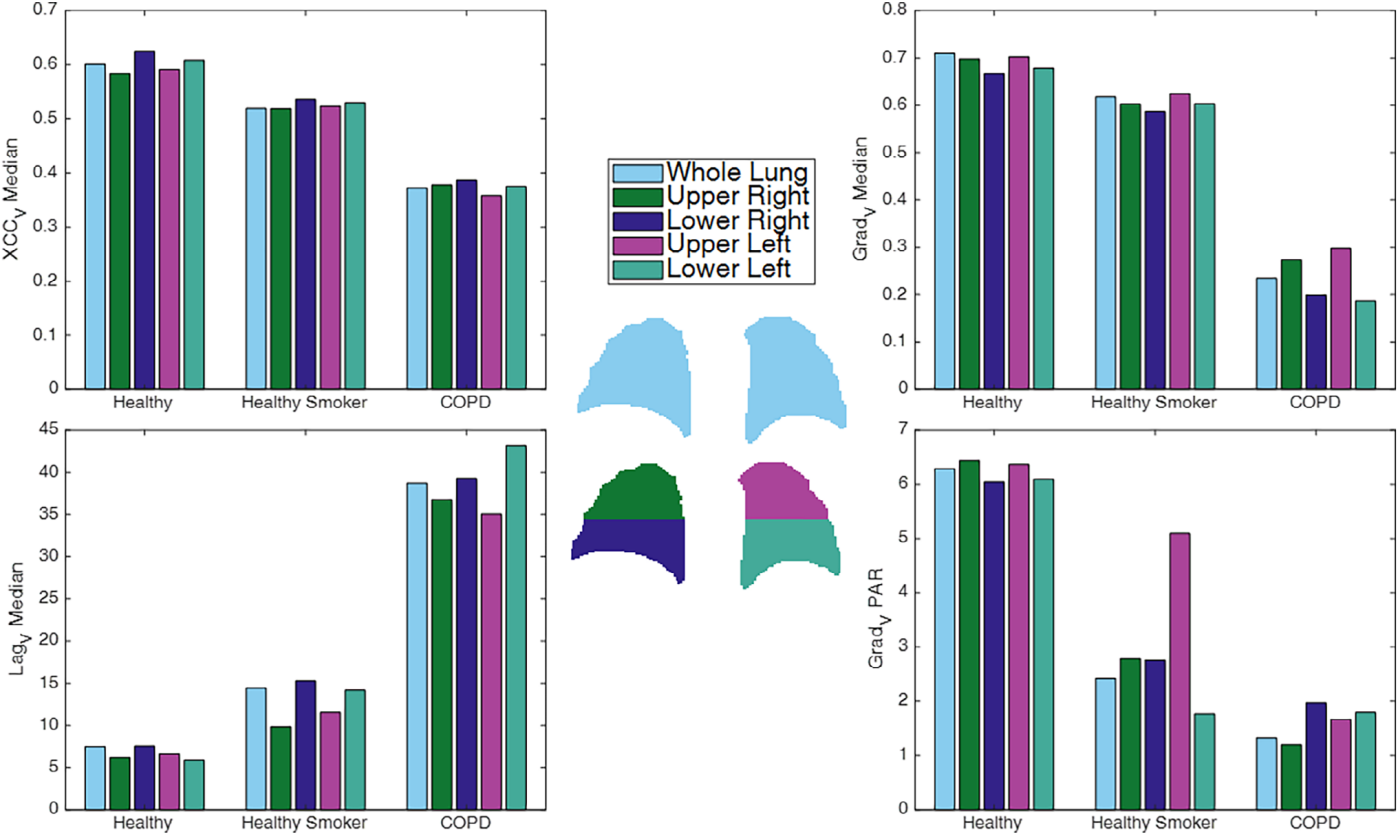
VOLVE metrics shown for different regions across the lung from the quadrant segmentation. Note that left/right refer to anatomical (rather than image) orientation. XCC = cross‐correlation; grad = gradient; PAR = peak area ratio.

Figure 6 shows that in both healthy participant groups there was little variation in VOLVE metrics across lung regions (except healthy smoker Grad_V_ PAR), whereas there tended to be greater relative regional variation in those with COPD, although no significant differences were found between quadrants within groups.

### VOLVE: Perfusion Assessment

**Figure 7:**
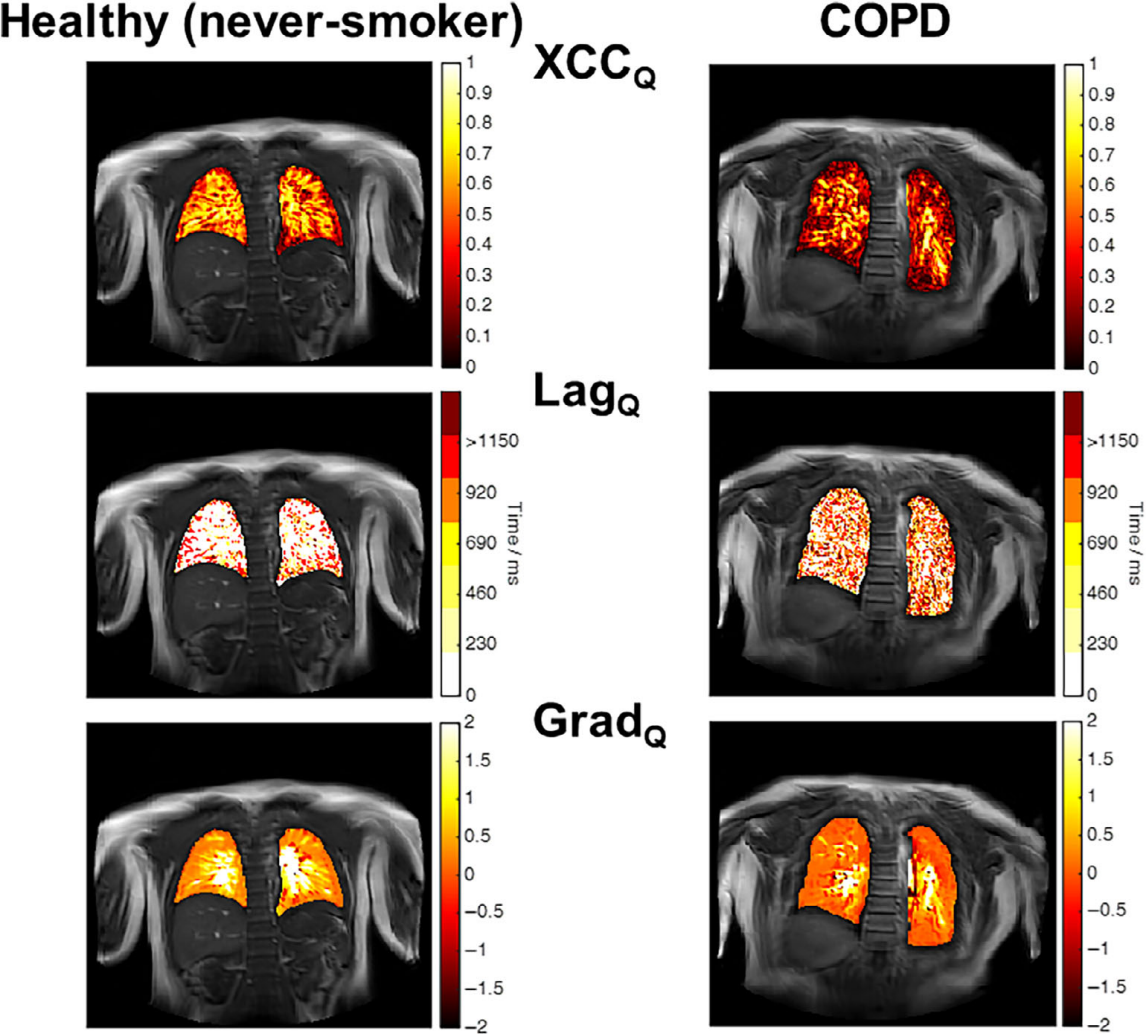
Example VOLVE‐based perfusion (Q) assessment maps. Cross‐correlation (XCC_Q_) maps (top row); lag time (Lag_Q_) maps (blood arrival time in milliseconds) (middle row); and gradient (Grad_Q_) maps (bottom row), for a healthy never‐smoker (age 23 years, female) and COPD participant (age 79 years, male, FEV_1_ = 61%, GOLD II).

**Figure 8:**
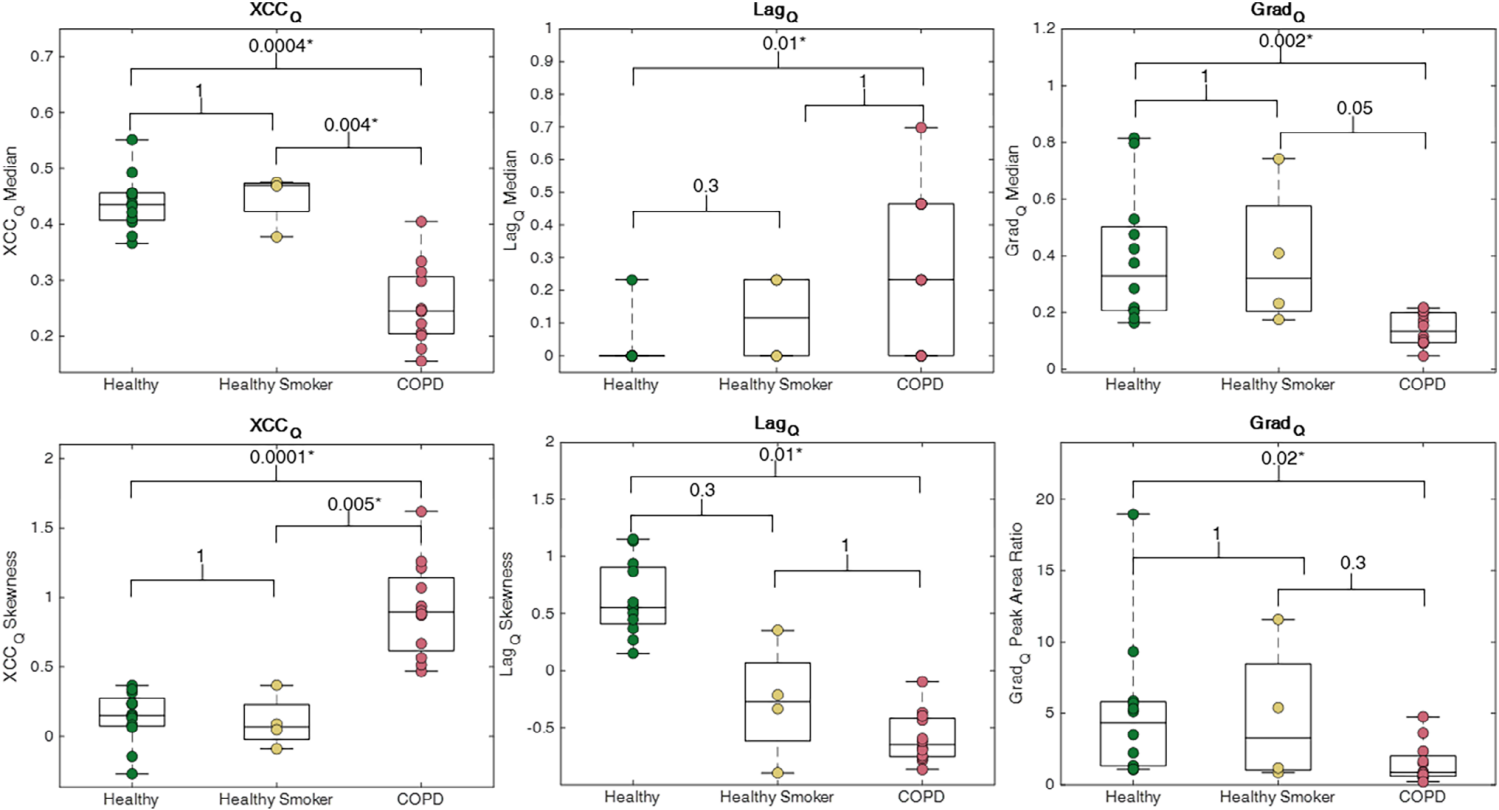
Boxplots showing the median and skewness for perfusion cross‐correlation (XCC_Q_) and lag (Lag_Q_), and median and fitted peak area ratio (PAR) for gradient (Grad_Q_). Significant differences (*P* < 0.05*, Kruskal–Wallis test with Dunn's post hoc analysis) between COPD patients and healthy participants (never‐smokers and those with smoking history) are observed for the XCC_Q_ metrics, and between COPD and healthy never‐smokers for Lag_Q_ and Grad_Q_ metrics.

Example VOLVE‐perfusion XCC_Q_, Lag_Q_ and Grad_Q_ maps are shown in Fig. 7. Strong XCC_Q_ are observed across the entire lung in the healthy never‐smoker participant, whereas weaker XCC_Q_ are seen in the COPD participant. Grad_Q_ values show a similar trend, with particularly strong Grad_Q_ observed in the main pulmonary vessels. All perfusion metrics were significantly different in COPD compared to healthy never‐smokers, but only the XCC_Q_ was significantly different between healthy‐smokers and COPD (Fig. 8).

## Discussion

In this study we described VOLVE, a novel analysis of widely available free‐breathing FFE MRI data to assess pulmonary ventilation and perfusion using standard MRI equipment. We demonstrated the feasibility of using linear postprocessing that exploits the full respiratory lung signal time course to determine lung ventilation‐related information. The method is repeatable (within‐visit, without repositioning participant) and robust as it uses automated analysis to assess lung parenchyma signals from across the entire respiratory cycle of multiple respiratory cycles. This method builds on previous approaches^2^, ^7^, ^9^ by introducing additional metrics that better describe and link the MR signal response to the physiological processes which drive the signal variations in the lung parenchyma. This enables better understanding of the data and may enable additional granularity of functional assessment. Additionally, no sorting or temporal interpolation is required which is advantageous in situations of low signal‐to‐noise ratio (SNR) such as the hyperinflated lung at 3 T.

### Ventilation Metrics in Different Participant Groups

Significant differences were observed in the Grad_V_, XCC_V_, and Lag_V_ (all VOLVE metrics) between COPD participants and the healthy never‐smoker group, and these differences may help to elucidate the physical relevance of the metrics. The cross‐correlation, XCC_V_, demonstrates the extent to which the lung parenchyma signal covaries with the navigator signal (and thus lung volume), independent of any lag in response. In the healthy lung, we observed a strong positive cross‐correlation, minimal lag and positive gradient, as the alveoli expand and contract with the respiratory cycle, giving rise to the expected tissue density and corresponding MR signal changes. The gradient, Grad_V_, describes the magnitude of the MR signal change with lung volume and characterizes the fundamental MR response to respiration; it is analogous to FV but uses data from the entire respiratory cycle.

Participants with COPD demonstrated an altered VOLVE response. We found that in COPD, a greater proportion of voxels exhibited lower cross‐correlation indicating a reduction in the linearity of the response. We believe that these are areas of alveolar hypoventilation which experience little or no variation in proton density during the respiratory cycle. Similar sensitivity to hypoventilation in COPD, and delineation from healthy groups, has been shown recently with the PREFUL Vent‐CC metric.^25^, ^26^

We also observed an increase in the number of voxels with longer lag and negative gradient, suggesting asynchrony or inversion of the expected relationship between the lung parenchymal signal and diaphragm navigator signal. Regions demonstrating large lags and negative gradients (particularly observed in participants with FEV_1_ < 50% but not for those with milder airflow obstruction) may be indicative of delayed or paradoxical ventilation due to expiratory flow limitation.^27^, ^28^ This may reflect severe small airways obstruction, where limitation of expiratory airflow leads either to air trapping or diversion of airflow through collateral channels. The Lag_V_ may reflect similar information to the PREFUL ventilation time‐to‐peak parameter, which was shown to be heterogeneous in COPD.^25^

A simpler VOLVE metric is the Pearson correlation coefficient (CC_V_, corresponding to XCC_V_ for zero lag), which provided excellent delineation between the groups, owing to the increased lag observed in COPD that increased the difference in the CC_V_ (see Supplementary Information S4). However, CC_V_ cannot distinguish voxels with a lagged response from those that do not exhibit a linear variation with lung volume, whereas the XCC_V_ can, thus probing the fundamental underlying signal variations. Further work is needed to investigate voxels with nonlinear responses which may be due to hysteresis (i.e., the volume change with respect to navigator signal changes between inspiration and expiration^29^).

### Repeatability of Ventilation Metrics

The intraparticipant test–retest repeatability was assessed with no repositioning of the participants. The Grad_V_ PAR VOLVE metric showed particularly narrow limits of agreement (−0.384 to 0.306) relative to the differences between the groups (3.9 between healthy never‐smoker and COPD). The observed CoV of generally <12% for the ventilation metrics is similar to other recent studies using the PREFUL method.^25^, ^30^

Lower repeatability in VOLVE metrics was observed overall in the COPD group. This may be due to the minus pathologies (reduction in tissue density) associated with COPD reducing the available SNR.^31^ The higher CoV values in the COPD group for Lag_V_ could be in part be due to the lag distribution being multimodal in some cases and hence poorly characterized by the median. Since the repeat scans were collected in the same examination, changes are unlikely to be due to genuine changes in lung function. Repeatability in COPD participants may also be impacted by prolonged supine posturing and compression of the thorax by the MRI chest coil during scanning, particularly during the approximate 15 minutes between the two scans, which may have led to progressive dynamic hyperinflation, although no systematic change was observed. Repeatability may be impacted by technical factors such as participant motion between scans or imperfect registration, and could potentially be improved with the implementation of a deep‐learning segmentation of lung parenchyma^32^ in the analysis pipeline.

### Regional Analysis of Ventilation

There tended to be relatively greater regional variation in VOLVE ventilation metrics in COPD than healthy participants, as is expected for this heterogeneous disease, although no significant differences were found between lung quadrants within groups. This type of regional analysis has potential to provide clinically relevant information in cases of targeted interventions such as lung volume reduction procedures.

### Comparison of Ventilation Metrics With Spirometry

We observed moderate but significant relationships between spirometry measures (% predicted FEV_1_ and FEV_1_/FVC) and XCC_V_ and Grad_V_ metrics across the healthy smoker and COPD groups. VOLVE and other MRI ventilation measures may not exhibit strong correlation with spirometry because these methods probe different aspects of lung physiology. MRI methods are sensitive to the presence/absence of ventilation, even at the level of the smallest airways/alveoli, whereas spirometry is predominantly a measure of larger airway function. For instance, it is well established that substantial small airways disease must be present before FEV_1_ values are abnormal,^33^ whereas VOLVE is potentially more likely to be sensitive to mild disease.

### Perfusion Assessment

The Lag_Q_ (blood arrival time) map for healthy participants generally showed a short blood arrival time, indicating that the lung was well‐perfused. In contrast, COPD participants exhibited longer blood arrival times and greater heterogeneity. Significant differences between COPD and healthy never‐smokers for all VOLVE‐perfusion metrics illustrate the sensitivity to perfusion defects and provide the possibility of visualizing ventilation/perfusion (V/Q) match and mismatch.

An advantage of the VOLVE approach is that it is possible to separate the time series into both periods of inspiration and expiration, and periods of diastole and systole. This may enable VOLVE analysis of cardiac‐gated ventilation metrics and respiratory‐gated perfusion metrics. We undertook cardiac‐gated ventilation analysis on this data (Supplementary Information S5) and no significant differences for ventilation were found (compared to the un‐gated approach) in these groups of participants; however, it may be that such an approach will be useful in some clinical scenarios such as in patients with cardiac arrhythmia.

### Limitations

A drawback of the histogram analysis is that data is compressed, such that regional maps are reduced to global parameters. Other quantitative metrics could be explored in future which preserve some regional information, to quantify heterogeneity and distribution of V/Q defects.

The pilot study had a small sample size, and the groups were not age‐matched, which may contribute to some differences between the groups. The healthy smoker group was particularly small. A future study should establish quantitative benchmark values in healthy never‐smokers to enable the generation of binary defect maps and ventilation defect percentage to facilitate rapid clinical interpretation. The VOLVE response in diseases other than COPD may be different, particularly if diaphragm dynamics are differently affected, or disease manifests as an increase in proton density e.g., fibrosis, and so future studies should consider participants with a range of other respiratory diseases.

A limitation of this study was that only retrospective spirometry data was available, which may not have accurately reflected contemporaneous lung function. This was due to local COVID‐19 safety measures during the initial period of data acquisition which forbade the performance of aerosol‐generating procedures (this is also the reason for the lack of spirometry data for healthy never‐smokers).

VOLVE has not been compared to other established methods such as PREFUL because a fair comparison would require access to proprietary software. Future studies should compare VOLVE to other contemporary methods to investigate any potential differences in robustness, sensitivity, and repeatability.

This study assumes that the navigator signal is an indicator of lung volume. However, it is not a measure of absolute lung volume and indeed the relationship between navigator signal (generated from the right lung–diaphragm boundary) and total lung volume may become nonlinear, for example, if there is a change in interplay between the diaphragm and the chest wall, or asymmetric diaphragm excursion. An alternative approach would be to measure the relationship between navigator signal and lung volume, hence allowing parenchyma signals to be compared to lung volume changes. This would allow more direct quantification of the relationship between parenchyma density and lung volume.^29^

As with other studies using a similar acquisition,^30^, ^34^ a principal weakness of this approach is that only a single slice is studied. Increased volume coverage which is key for clinical translation could be achieved in future, either by reducing the length of the dynamic data series and scanning different slices consecutively, or by decreasing the dynamic rate and collecting interleaved slices concurrently, or by using a 3D acquisition.^17^, ^32^, ^35^, ^36^ The trade‐offs between temporal and spatial undersampling, and the effects of saturation of inflowing blood on the perfusion signal in these different imaging strategies requires further investigation. In addition, the optimum flip angle for obtaining a strong ventilation and perfusion signal may be different (lower) than the chosen value of *α* = 18°—further work is required to determine the flip angle for optimum sensitivity to both measures.^22^

This study was conducted at 3 T, and so the methodology presented requires validation at 1.5 T for wider translation. Previous studies have found good agreement between V/Q measures and SNR at 1.5 T and 3 T.^37^, ^38^, ^39^ While it is not expected that the SNR in the navigator signal will be substantially different at 1.5 T, the change in T_2_* could increase the signal and change the sensitivity to different lung pathology.

## Conclusion

VOLVE is a novel method to assess ventilation and perfusion in the lung from free‐breathing ^1^H MRI. The linear regression‐based approach utilizes all images in the respiratory cycle and does not require assumptions be made about the data. VOLVE metrics enabled healthy participants to be discerned from patients with COPD.

## Supporting information


**Data S1** Supporting information.
