# Supplementary material for: Free‐Breathing Functional Pulmonary Proton MRI: A Novel Approach Using Voxel‐Wise Lung Ventilation (VOLVE) Assessment in Healthy Volunteers and Patients With Chronic Obstructive Pulmonary Disease
Source: J Magn Reson Imaging. 2024 May 31;61(2):663–75. doi: 10.1002/jmri.29444 (PMC11706312; doi:10.1002/jmri.29444)
Supplement: Supplementary file 1 — Data S1 Supporting information. [file JMRI-61-663-s001.docx]

**Supplementary Information**

S1: York Regression

A simulation is shown in Figure S1 that demonstrates the effect of phase shift on the determined gradient for ordinary least-squares (OLS) regression and for York regression (YR). For a phase difference of up to ± π/2, the York regression can be seen to be robust in determining the gradient. For a phase difference of more than π/2 (but less than 3π/2), York regression finds a negative gradient. Hence, we can determine magnitude signal changes of the lung parenchyma relative to the navigator signal that are robust to phase shifts between the signals. Due to the step-like behaviour at nπ/2, some lung voxels (with a lag of 24.5-25.5%) have been removed from the lung mask to combat any adverse effect on the determined gradient maps.


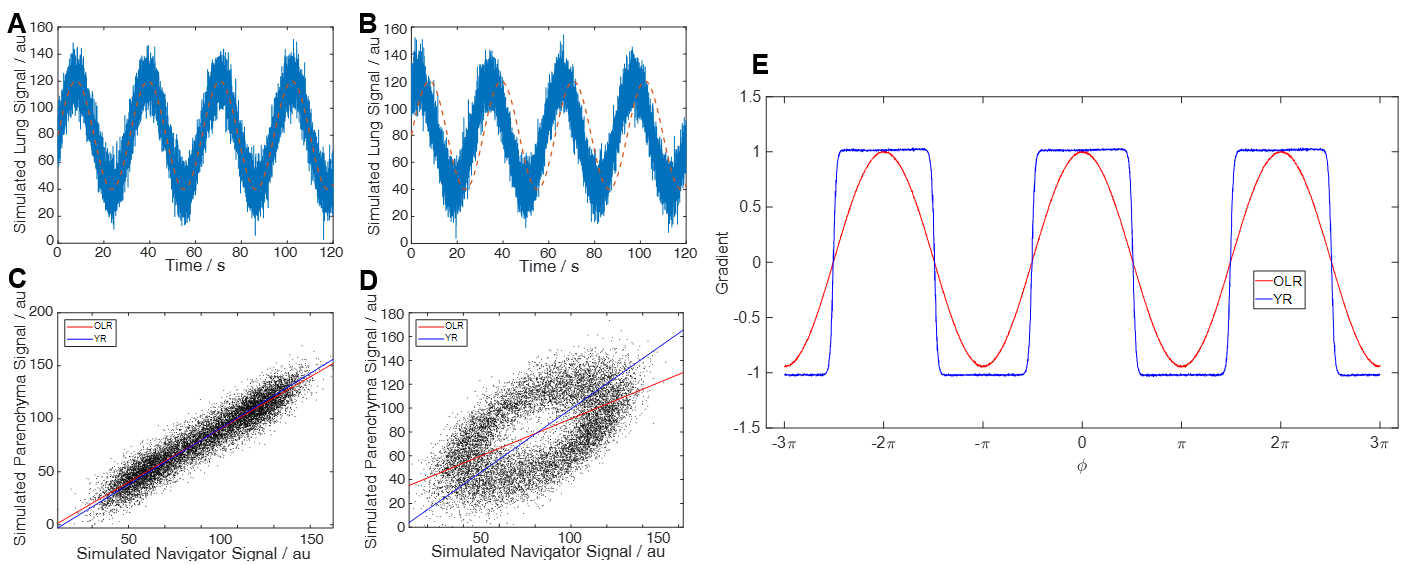


Figure S1: Simulated respiratory timecourse with Gaussian noise, without (**A**) and with (**B**) a phase shift. (**C, D**) shows ordinary linear regression (OLR) and York regression (YR) in (**A, B**), with N=10000 simulated Y signals (simulated lung parenchyma signals) and N=1 X signals (simulated navigator signal). Note that for this phase shift (π/3 or 60°), the OLR gradient is shallower, while the YR gradient remains the same. (**E**) shows the relationship between gradient and phase for OLR and YR.

S2: All Participant Maps


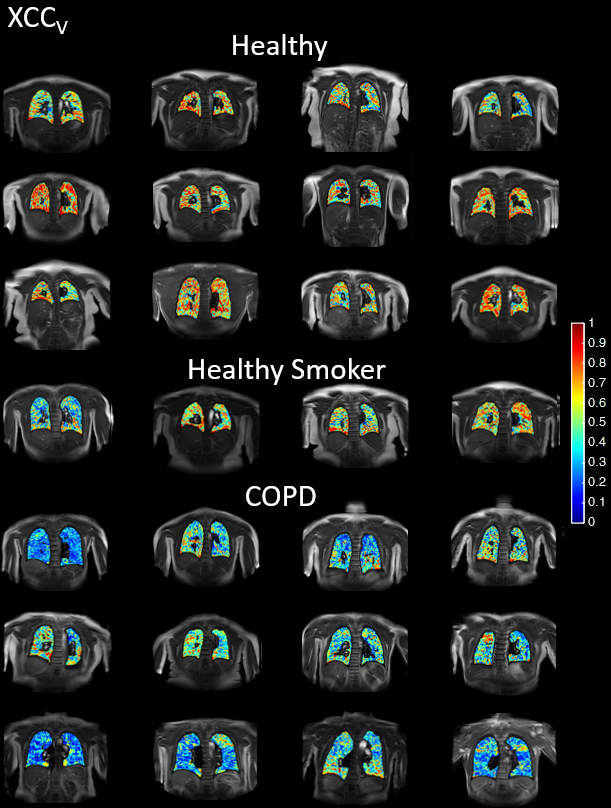


Figure S2.1: All participant maps for VOLVE ventilation: cross-correlation (XCC_V_).


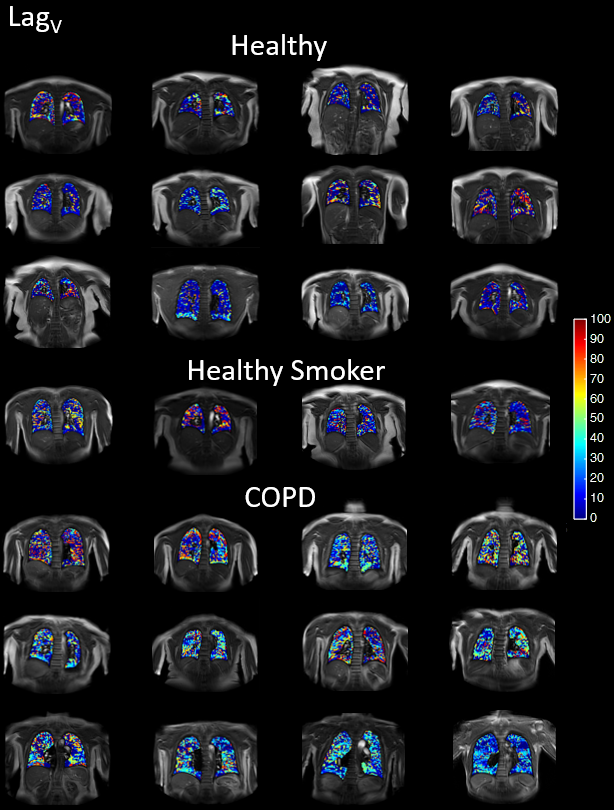


Figure S2.2: All participant maps for VOLVE ventilation: Lag_V_.


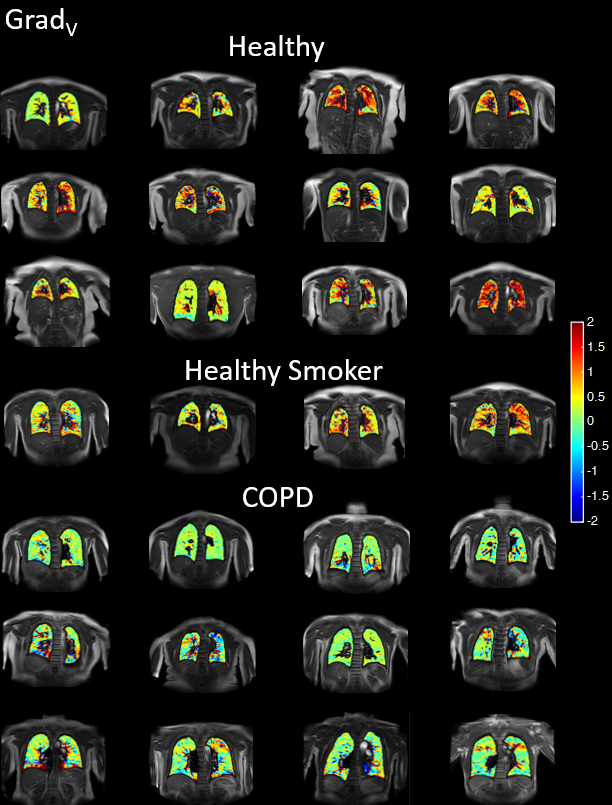


Figure S2.3: All participant maps for VOLVE ventilation: York gradient (Grad_V_).

S3: Repeatability Results

| **Metric** | **Repeat 1** | **Repeat 2** | **p** | **CoV (%)** |
| --- | --- | --- | --- | --- |
| **Healthy (never-smoker)** | | | | |
| **XCC_V_ Median** | 0.59 (0.55–0.66) | 0.59 (0.55–0.64) | 0.5693 | 5.16 |
| **XCC_V_ Skewness** | -0.34 (-0.58– -0.28) | -0.36 (-0.61– -0.27) | 0.9697 | 24.04 |
| **Lag_V_ Median** | 1.79 (0.00–4.85) | 1.88 (0.00–5.35) | 0.4375 | 6.15 |
| **Lag_V_ Skewness** | 0.86 (0.56–1.10) | 0.95 (0.54–1.19) | 0.3013 | 27.51 |
| **Grad_V_ Median** | 0.65 (0.52–0.78) | 0.67 (0.50–0.78) | 0.4238 | 14.60 |
| **Grad_V_ PAR** | 5.40 (4.47–7.71) | 5.20 (4.67–7.61) | 0.5186 | 2.23 |
| **Healthy (smoking history)** | | | | |
| **XCC_V_ Median** | 0.55 (0.47–0.59) | 0.60 (0.49–0.62) | 0.2500 | 4.89 |
| **XCC_V_ Skewness** | -0.22 (-0.38–0.03) | -0.34 (-0.42– -0.11) | 0.2500 | 29.67 |
| **Lag_V_ Median** | 7.14 (6.64–7.34) | 6.80 (6.21–7.14) | 0.6250 | 9.81 |
| **Lag_V_ Skewness** | 0.54 (0.35–0.69) | 0.62 (0.38–1.03) | 0.3750 | 34.39 |
| **Grad_V_ Median** | 0.59 (0.51–0.70) | 0.62 (0.51–0.78) | 0.3750 | 12.43 |
| **Grad_V_ PAR** | 4.93 (3.50–5.74) | 5.01 (3.63–5.65) | 0.8750 | 2.94 |
| **COPD** | | | | |
| **XCC_V_ Median** | 0.40 (0.31–0.43) | 0.34 (0.27–0.38) | 0.1161 | 11.59 |
| **XCC_V_ Skewness** | 0.29 (0.20–0.54) | 0.34 (0.25–0.45) | 0.9697 | 35.87 |
| **Lag_V_ Median** | 23.24 (21.41–27.07) | 21.13 (18.91–25.72) | 0.2061 | 11.97 |
| **Lag_V_ Skewness** | -0.09 (-0.62–0.12) | -0.31 (-0.86– -0.06) | 0.3804 | 5.02 |
| **Grad_V_ Median** | 0.20 (0.16–0.24) | 0.27 (0.20–0.36) | 0.1884 | 20.54 |
| **Grad_V_ PAR** | 1.49 (0.95–1.70) | 1.50 (1.08–1.89) | 0.1210 | 9.74 |

Table S.3: repeatability assessment. Repeat values quoted as median (25^th^-75^th^ percentile), p values from Wilcoxon signed-rank test, CoV = coefficient of variation).

S4: Correlation Coefficient

As an alternative to using the cross-correlation (XCC_V_), lag (Lag_V_) and gradient (Grad_V_) to characterise the local parenchyma signal changes with respect to the global navigator signal, a simplified approach involves using the Pearson correlation coefficient (CC_V_) which reflects both XCC_V_ and Lag_V_. The CC_V_ demonstrates the extent to which the signals from individual lung voxels covary in-phase with the navigator. Example maps and histograms are shown in Figure S.4.1. The CC_V_ map for the healthy participant is relatively homogenous with CC_V_ values > 0.7 (histogram negatively skewed; skewness = -1.44), except for some small regions in, or bordering, vessels. In contrast, the CC_V_ map for the COPD participant is heterogeneous (histogram symmetrically distributed with a skewness = -0.04), with a substantial proportion of lung parenchyma voxels displaying a CC_V_ of 0 or less, a lower correlation compared to the XCC_V_ values, where a negative correlation coefficient indicates an *increase* in MR parenchyma signal on inspiration. In this case the CC_V_ is picking up those voxels that have a large lag/phase shift as well as magnitude change.

Figure S.4.2 shows boxplots of the CC_V_ median and skewness which were significantly different between the healthy never-smoker and COPD groups.

The XCC_V_ is an informative measure as the quantification of the delay (lags) along with the linearity (XCC) and magnitude (gradient) of the response provides a holistic description of the MR signal variations of the lung parenchyma. However, the CC effectively combines the information available in the XCC and lag and provides a metric which gives clear delineation between the groups.


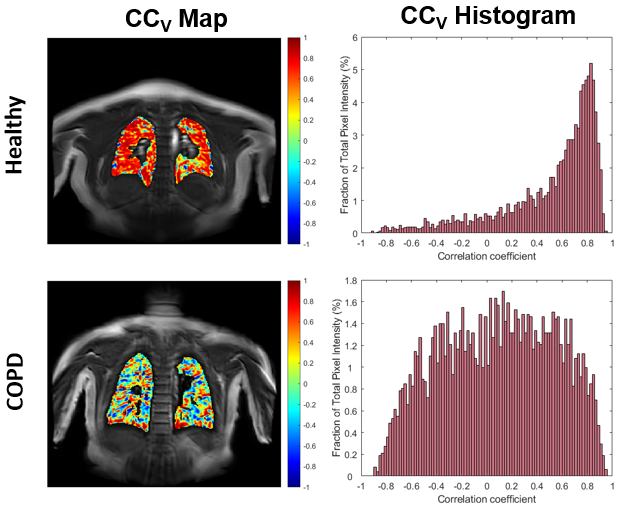


Figure S.4.1: Example correlation coefficient (CC) map and histogram for a healthy never-smoker (age 24 years, male) and COPD participant (age 66 years, female, FEV_1_=30%, GOLD III).


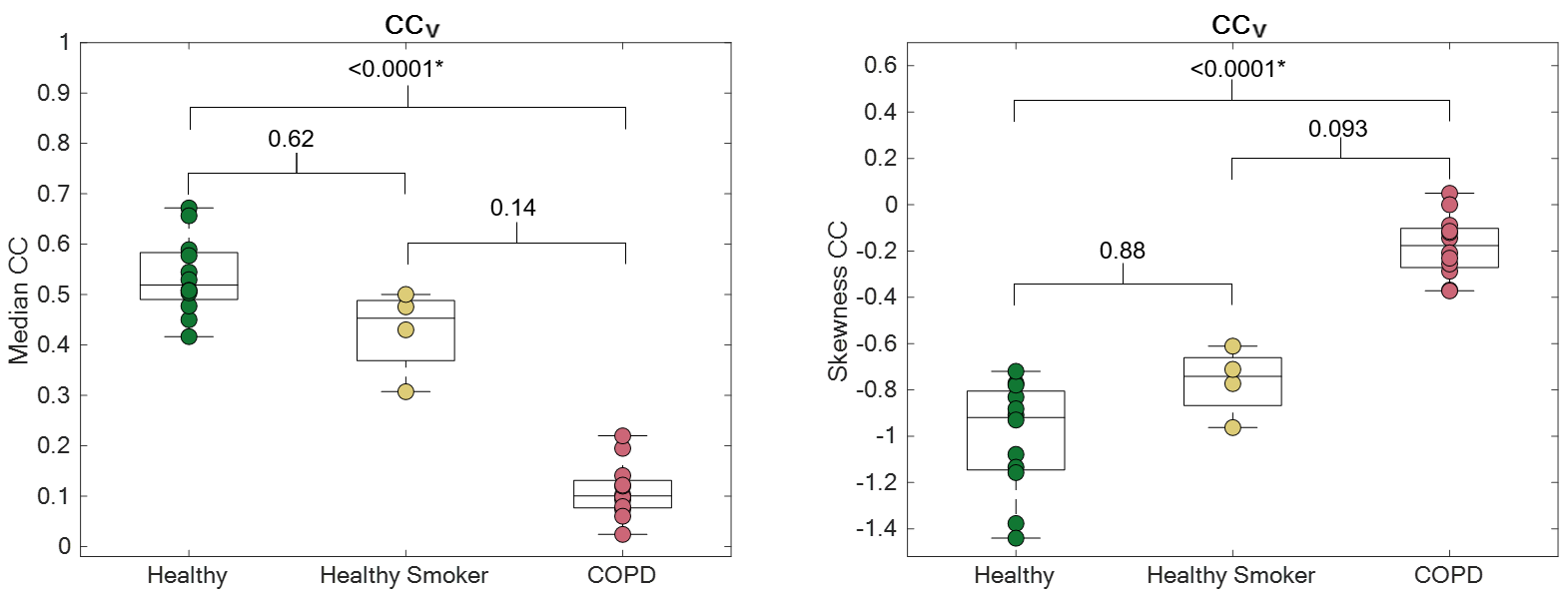


Figure S.4.2: Boxplots showing correlation coefficient (CC_V_) median and skewness for the groups. Significant differences (p<0.05, Kruskal-Wallis test with Dunn’s post-hoc analysis) between the groups is observed for both metrics; significant results are marked with an asterisk.

S5: Cardiac-gated Ventilation

The cardiac signal timecourse derived from the aorta/ main pulmonary vessel ROI characterizes the cardiac phase of each image in the timeseries. This was used to compute VOLVE perfusion metrics (XCC_Q_, Lag_Q_ and Grad_Q_). The lung-diaphragm navigator signal characterises the respiratory phase of each image, and therefore both the respiratory and cardiac phase of each image is known.

The cardiac timecourse can therefore also be used to separate the ventilation maps into different cardiac phases, such that they can be reconstructed at systole and diastole (or perfusion maps reconstructed for inspiration and expiration) by subdividing the images accordingly.

Retrospective cardiac-gated ventilation maps were determined by subdividing the data set into systole and diastole by thresholding the cardiac signal timecourse at 80% and 20% of the maximum value, respectively, and performing VOLVE analysis on each time series separately.

Table S.5 shows VOLVE metrics with and without retrospective cardiac gating. all ventilation metrics that were significant prior to cardiac gating remain significant for COPD when gated to systole and diastole, except systole XCC_V_ median. There was a trend for VOLVE metrics determined at diastole to be closer to values obtained without cardiac gating, which could be due to reduced blood signal component (and so reduced cardiac ‘noise’) at diastole compared to systole.

| **Ventilation Metric** | **Healthy (Never Smoker)** | **Healthy (Smoking History)** | **COPD** | **Healthy never smoker vs. smoking history**  **p-value** | **Healthy never smoker vs. COPD p-value** | **Healthy smoking history and COPD p-value** |
| --- | --- | --- | --- | --- | --- | --- |
| **Ventilation Metrics (All Data)** | | | | | | |
| **XCC_V_ Median** | 0.61 ± 0.07 | 0.53 ± 0.092 | 0.38 ± 0.08 | 0.83 | <0.0001* | 0.15 |
| **XCC_V_ Skewness** | -0.42 ± 0.24 | -0.18 ± 0.31 | 0.34 ± 0.22 | 0.72 | <0.0001* | 0.17 |
| **Lag_V_ Median** | 2.45 ± 2.70 | 6.99 ± 0.59 | 24.75 ± 6.59 | 0.31 | <0.0001* | 0.24 |
| **Grad_V_ Median** | 0.68 ± 0.26 | 0.60 ± 0.13 | 0.21 ± 0.06 | 1 | <0.0001* | 0.02* |
| **Grad_V_ PAR** | 6.02 ± 1.93 | 4.62 ± 1.39 | 1.34 ± 0.67 | 1 | <0.0001* | 0.03* |
| **Cardiac-gated Ventilation Metrics** | | | | | | |
| **Diastole XCC_V_ Median** | 0.58 ± 0.12 | 0.478 ± 0.14 | 0.36 ± 0.10 | 0.90 | 0.002* | 0.5 |
| **Diastole XCC_V_ Skewness** | -0.33 ± 0.34 | 0.03 ± 0.48 | 0.40 ± 0.30 | 0.62 | 0.0005* | 0.5 |
| **Diastole Lag_V_ Median** | 0.42 ± 0.52 | 1 ± 0.82 | 3.58 ± 2.23 | 1 | <0.0001* | 0.14 |
| **Diastole Grad_V_ Median** | 0.61 ± 0.26 | 0.457 ± 0.13 | 0.19 ± 0.07 | 1 | <0.0001* | 0.04* |
| **Diastole Grad_V_ PAR** | 5.73 ± 2.14 | 3.872 ± 2.26 | 1.17 ± 0.60 | 1 | <0.0001* | 0.03* |
| **Systole XCC_V_ Median** | 0.44 ± 0.15 | 0.427 ± 0.16 | 0.32 ± 0.09 | 1 | 0.21 | 0.85 |
| **Systole XCC_V_ Skewness** | 0.056 ± 0.37 | 0.159 ± 0.45 | 0.44 ± 0.21 | 1 | 0.04* | 0.56 |
| **Systole Lag_V_ Median** | 1.92 ± 6.02 | 0.5 ± 0.58 | 3.67 ± 2.96 | 1 | 0.0007* | 0.05 |
| **Systole Grad_V_ Median** | 0.52 ± 0.23 | 0.457 ± 0.15 | 0.20 ± 0.07 | 1 | 0.0002* | 0.02* |
| **Systole Grad_V_ PAR** | 4.95 ± 1.88 | 5.167 ± 2.13 | 1.53 ± 0.69 | 1 | 0.0009* | 0.02* |

Table S.5: MRI Ventilation metrics (VOLVE cross-correlation (XCC_V_), lag (Lag_V_) and gradient (Grad_V_) with and without retrospective cardiac gating. Values quoted as mean ± standard deviation. Significant results (p<0.05) are marked with an asterisk.
